# The effect of lifestyle and risk factor modification on occlusive peripheral arterial disease outcomes: standard healthcare vs structured programme—for a randomised controlled trial protocol

**DOI:** 10.1186/s13063-021-05087-x

**Published:** 2021-02-13

**Authors:** M. Elfghi, F. Jordan, D. Dunne, I. Gibson, J. Jones, G. Flaherty, S. Sultan, W. Tawfick

**Affiliations:** 1 School of Medicine, National University of Ireland, University Road, Galway, Ireland; 2 School of Nursing and Midwifery, National University of Ireland, University Road, Galway, Ireland; 3 National Institute for Prevention and Cardiovascular Health, Croi Heart and Stroke Centre, Mayola Lane, Newcastle, Galway, Ireland; 4 Brunel University, Kingston Ln, Uxbridge, London UB8 3PH UK; 5 Department of Vascular and Endovascular Surgery, University College Hospital, Galway (UCHG), Newcastle Road, Galway, Ireland

**Keywords:** Peripheral arterial disease, PAD, Atherosclerosis, Risk factors, Lifestyle and risk factor modification intervention programme, Randomised controlled trial

## Abstract

**Background:**

Peripheral arterial disease (PAD) affects more than 200 million of the global population. PAD represents a marker for premature cardiovascular events. Patients with PAD, even in the absence of a history of myocardial infarction or ischemic stroke, have approximately the same relative risk of death from cardiovascular causes as patients with a history of coronary or cerebrovascular disease. Despite the high prevalence of PAD and the strong association with cardiovascular morbidity and mortality, patients with PAD are less likely to receive appropriate treatment for their atherosclerotic risk factors than those who are being treated for coronary artery disease.

Atherosclerotic risk factor identification and modification play an important role in reducing the number of adverse outcomes among patients with atherosclerosis. Risk reduction therapy decreases the risk of cardiovascular mortality and morbidity in patients with PAD. In this study, we aim to evaluate the effectiveness of a lifestyle and risk factor modification intervention programme in achieving treatment goals for PAD risk factors.

**Methods:**

This is a randomised, parallel group, active-control trial to compare the effectiveness of the risk factor modification intervention programme to standard healthcare in a tertiary vascular care centre, in the reduction of modified risk factors in PAD patients. The primary outcome of this study is to evaluate the effectiveness of a lifestyle and risk factor modification intervention programme in achieving treatment goals for PAD risk factors at 3 and 12 months. The secondary outcomes are to compare the impact of the programme on clinical outcomes in PAD patients at 12 months. Secondary outcomes include amputation-free survival, clinical improvement, haemodynamic improvement, need for revascularisation procedures, outcomes of revascularisation procedures, changes in quality of life and the incidence of adverse events.

**Discussion:**

This study will provide clear evidence on the effectiveness of a lifestyle and risk factor modification intervention programme in achieving treatment goals for PAD risk factors, through a high-quality, well-powered clinical trial.

**Trial registration:**

This trial was registered (11/07/2017) on the European Clinical Trials Database (EudraCT number 2017-002964-41) and ClinicalTrials.gov (NCT03935776) which was registered on 02 May 2019.

**Supplementary Information:**

The online version contains supplementary material available at 10.1186/s13063-021-05087-x.

## Background

Peripheral arterial disease (PAD) affects more than 200 million of the global population [1]. PAD represents a marker for premature cardiovascular events [2]. Patients with PAD, even in the absence of a history of myocardial infarction or ischemic stroke, have approximately the same relative risk of death from cardiovascular causes as patients with a history of coronary or cerebrovascular disease [2]. Despite the high prevalence of PAD and the strong association with cardiovascular morbidity and mortality, patients with PAD are less likely to receive appropriate treatment for their atherosclerotic risk factors than those who are being treated for coronary artery disease [3, 4].

As PAD represents a peripheral manifestation of atherosclerosis, most traditional and novel cardiovascular risk factors are strongly associated with this condition [5–7]. Smoking, diabetes, hyperlipidaemia, hypertension, unhealthy diet and physical inactivity were identified as significant modifiable risk factors that should be targeted for secondary prevention [8–12].

Atherosclerotic risk factor identification and modification play an important role in reducing the number of adverse outcomes among patients with atherosclerosis [13]. Risk reduction therapy decreases the risk of cardiovascular mortality and morbidity in patients with PAD [13]. Because of the efficacy of these techniques, several expert committees have recommended their use in patients with PAD [8–12]. Despite clear guidelines, several studies have shown that patients with PAD are routinely undertreated for these risk factors [4], which may contribute to high rates of morbidity and mortality.

Previous studies concluded that modifiable risk factor programmes help cardiac patients achieve their risk factor modification targets, with subsequent reduction in cardiovascular events [14–16]. To our knowledge, currently, there is still no evidence to support that the implementation of a structured modifiable risk factor reduction program will lead to improved outcomes in PAD patients. However, due to the similar atherosclerotic burden in cardiac and PAD patients, we hypothesise that these risk factor modification programmes would similarly improve risk factor target achievement in PAD patients and subsequently improve their clinical outcomes and reduce their amputation rates when compared to standard healthcare.

The possibility of lifestyle and risk factor modification intervention should have a positive impact on patients’ clinical well-being and on their quality of life.

## Study objectives

### Primary objective

The primary objective of this study is to evaluate the effectiveness of a lifestyle and risk factor modification intervention programme in achieving treatment goals for PAD risk factors compared to the standard healthcare management.

### Secondary objectives

Secondary objectives are to compare the impact of the programme on clinical outcomes in PAD patients, specifically:

- Amputation-free survival
- Clinical improvement [17]
- Haemodynamic improvement [17]
- Need for revascularisation procedures
- Changes in quality of life [18]
- The incidence of adverse events

## Methods

### Study design

This is a randomised, parallel group, active-control trial to compare the effectiveness of the risk factor modification intervention programme to standard healthcare in a tertiary vascular care centre, in the reduction of the prevalence of modifiable risk factors in PAD patients.

**Fig. 1:**
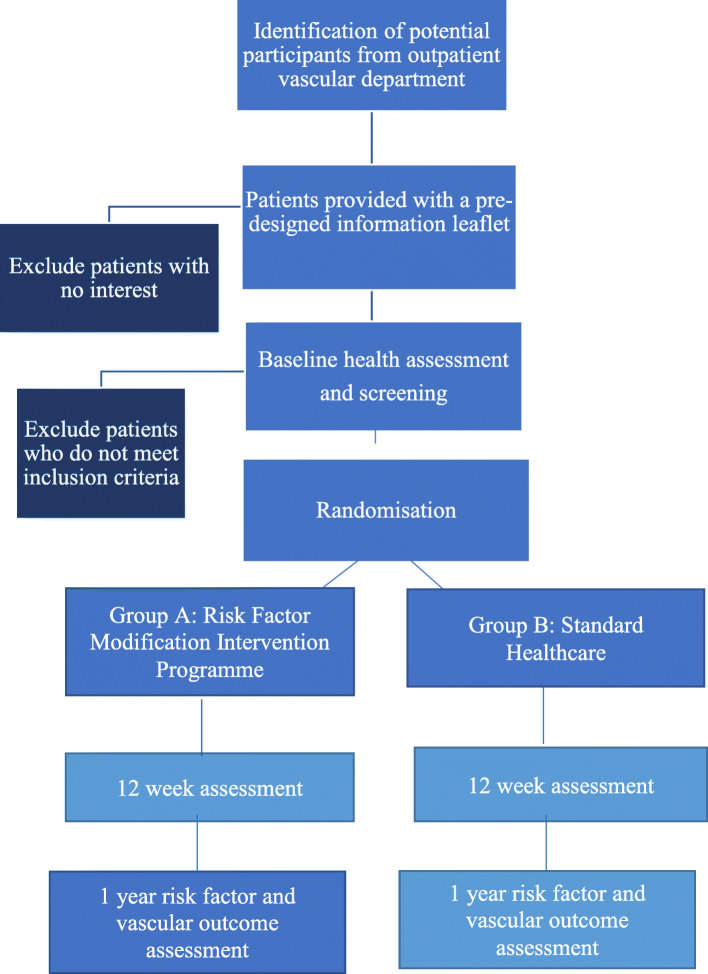
Participant flowchart through the study

**Fig. 2:**
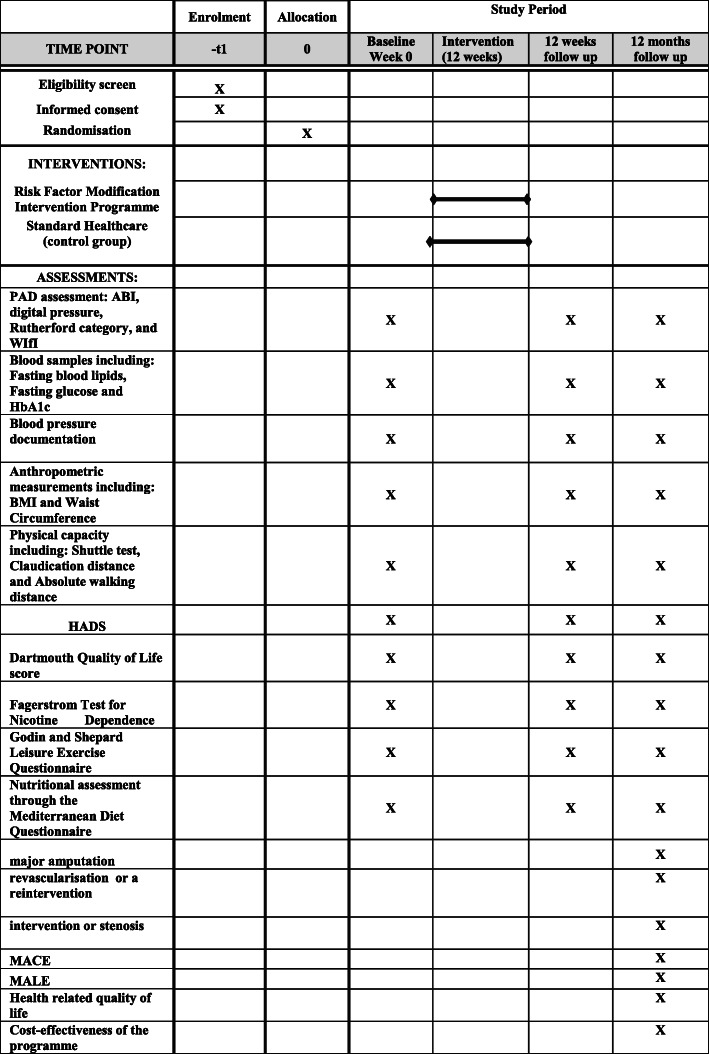
SPIRIT figure showing an overview of the assessment schedule at baseline and follow-up in study. ABI: ankle-brachial Index, WIfI: wound ischaemia and foot infection classification, HbA1c: glycosylated haemoglobin, BMI: body mass index, HADS: Hospital Anxiety and Depression Scale, MACE: major adverse cardiovascular event and MALE: major adverse limb event

This trial will randomise patients with PAD in an equal ratio to one of two treatment arms. One arm will be randomised to undergo a risk factor modification intervention programme at a community-based centre. The other arm will be provided with standard healthcare advice, in the outpatient PAD clinic in a tertiary referral vascular centre. Patients randomised into the risk factor modification intervention programme will have the intervention administered for 12 weeks. Patients will then be followed for 12 months to determine further PAD outcomes. The participant flowchart through the study is shown in Fig. 1. SPIRIT figure for this trial is given in Fig. 2.

### Study setting

Potential participants will be identified from the outpatient PAD clinic at the University Hospital Galway, Ireland (UHG). Patients will be screened and randomised at the outpatient PAD clinic in UHG.

The risk factor modification intervention programme will be administered in a nurse-led community-based centre (Croí Heart and Stroke Centre, Galway, Ireland), in the presence of a physiotherapist and dietitian. The control arm will be managed in the outpatient PAD clinic, UHG. The coordinating centre will be the Department of Vascular and Endovascular Surgery, UHG, and the School of Medicine at the National University of Ireland Galway (NUI Galway). Patients are directly supervised during the intervention. The 12-week and 1-year assessments will take place at the outpatient PAD clinic in UHG.

### Eligibility criteria

#### Inclusion criteria

- Aged 18 years or more
- Provide written informed consent
- PAD: diagnosed by at least one of the following:
  - Ankle-brachial index of less than 0.90 in at least one lower extremity [9]
  - Toe brachial index of less than 0.60 [9]
  - Evidence of arterial occlusive disease in one lower extremity detected by duplex ultrasonography, computed tomographic angiography, or magnetic resonance angiography [9]
- Symptomatic PAD (Rutherford category 2 and above [19])
- Patients should have at least one of the following risk factors:
  - Blood pressure > 140/80 mmHg
  - Fasting blood sugar (FBS) > 53 mmol/mol
  - Glycosylated haemoglobin (HbA1c) > 7%
  - Total cholesterol > 5 mmol/L
  - Low-density lipoprotein (LDL) cholesterol > 2.6 mmol/L
  - Triglycerides > 1.7 mmol/L
  - High-density lipoprotein (HDL) < 1.0 mmol/L in men and < 1.2 mmol/L in women
  - Physical activity less 30 min for 5 days per week
  - Body mass index (BMI) > 25 kg/m^2^
  - Waist circumference > 80 cm in women, and > 94 cm in men
  - Current smoker or exposure to tobacco in any form
  - Unhealthy diet, Mediterranean diet score less than 10 points

#### Exclusion criteria

- Involvement in another clinical trial in the previous 6 months
- Legal incapacity
- Inadequate English language ability to understand the content of the intervention programme
- Significant cognitive impairment or mental illness
- Refusal to participate in a certain part of the intervention
- Patient suffering from a comorbidity that could affect either their physical participation in the intervention arm or influence outcomes (e.g. ischemic heart failure or severe chronic kidney disease with an estimated glomerular filtration rate of less than 30 mL/min [20])
- Patient is immobile
- Contraindication to anticoagulation and antiplatelet medications or any of the risk factors treatment.

### Study screening

Patients with Symptomatic PAD (Rutherford category 2 and above [19]) will be invited to join the study. Invited patients will be provided with a pre-designed information leaflet. This leaflet will be fully explained to the patient at the initial assessment. The study researchers will answer any questions about the study. Informed consent will be obtained from the patient on a formatted consent form. Patients will be given the freedom to give consent either on the same day or at a later date in accordance with a study within a trial (SWAT), entitled “Same-day Consent vs Delayed Consent in a Randomised Trial: A Study within a Trial” [21]. This SWAT aims to ensure the rigorousness of the consent process and will run in conjunction with this randomised controlled trial.

Researchers will screen the patient for inclusion and exclusion criteria and administer a series of the following:

1. Record PAD risk factors such as smoking, hyperlipidaemia, diabetes, hypertension, increased body weight and the patient’s current medication.
2. Document the Rutherford category [19], claudication distance and absolute walking distance for each patient, to assess the severity of PAD.
3. Schedule appointments for baseline health assessments over the following month.

### Randomisation

After meeting the inclusion criteria, screened patients will be randomised to one of two treatment arms. One arm will receive the 12-week intensive risk factor modification intervention programme. The control arm will be provided with standard care in the outpatient PAD clinic. This is an intention to treat designed study, where patients are analysed as randomised. Each screened patient will be given a unique screening number.

Screened patients will be randomised in a 1:1 ratio of study intervention: control according to a randomisation scheme. The randomisation scheme will be produced using the PROC PLAN® procedure of the SAS®software package (version 9.2.2) using a simple randomisation strategy. The scheme will be concealed from all patients and study personnel until after database lock.

Patients will be allocated to intervention via an automated telephone system, which will not deliver the randomised allocation except after registering the subject screening number. Each screened patient who is recruited to the trial will be given a unique patient trial number.

The statistician will remain blinded to the treatment allocation until all the data have been analysed to minimise bias. Outcome assessors and data analysts will be blinded; however, in the event of an adverse event outcome, assessors will be unblinded.

### Baseline, prior to intervention

All randomised patients will undergo a full baseline assessment prior to their intervention:

1. PAD assessment;
  - Ankle-brachial index (ABI)
  - Digital pressure
  - Rutherford category [19]
  - Wound ischaemia and foot infection classification (WIfI) [22]
2. Blood samples including:
  - Fasting blood lipids
  - Fasting glucose
  - HbA1c
3. Blood pressure documentation
4. Anthropometric measurements including:
  - BMI
  - Waist circumference
5. Sub-maximal functional capacity exercise testing including:
  - Shuttle test [23]
  - Claudication distance [24]
  - Absolute walking distance [25]
6. Behavioural and psychological survey using the Hospital Anxiety and Depression Scale (HADS) [26].
7. Health-related quality of life assessment: Dartmouth Quality of Life score [18].
8. Smoking status assessment using the Fagerstrom Test for Nicotine Dependence [27].
9. Physical activity assessment: Godin and Shepard Leisure Exercise Questionnaire [28].
10. Nutritional assessment through the Mediterranean Diet Questionnaire [29].

### Intervention

#### Risk factor modification structured Programme

The risk factor modification intervention programme is a 12-week intensive lifestyle programme. This is a nurse-led, community-based, lifestyle and risk factor modification intervention modelled on the European Society of Cardiology demonstration project, a large clinical trial called EuroAction [30]. The programme includes:

- Phase 1: Initial individualised assessment by the multidisciplinary team (MDT) will include previously mentioned baseline assessment in addition to the following:
  - Dietician will assess, current eating habits and food diary
  - Exercise specialist will assess 7-day activity recall, barriers to exercise, 7-day pedometer and Functional Capacity Test
- Phase 2: The intervention including:
  - Weekly exercise class and educational workshops.
  - Serial blood pressure, body mass index, waist circumference, glucose and lipid measurements with goal setting.
  - Weekly MDT meetings.
  - Targeted and protocol-based pharmacotherapy to support lifestyle changes.

#### Standard healthcare

The control group will receive the standard healthcare advice provided to PAD patients in the outpatient PAD clinic. In this study, standard care will be conducted by the researchers which include:

- Advising patients to quit smoking, regular exercise and healthy eating, but neither structured intervention nor organised cessation plans will be addressed.
- Non-specific interventions, such as providing patients with educational material on general health problems.

#### Twelve-week assessment

On completion of the 12 weeks in both groups, patients are reassessed for risk factors, therapeutic management and lifestyle changes similar to baseline.

#### One-year assessment

Similar to the baseline and 12-week assessment, in addition to clinical outcomes assessment, the following are included:

1. If the patient underwent a major amputation and level of amputation
2. If required a revascularisation procedure.
3. Any residual stenosis of more than 30% [17]
4. If developed a major adverse cardiovascular event (MACE) or major adverse limb event (MALE)
5. Health-related quality of life
6. Cost-effectiveness of the programme

### Endpoints

#### Primary endpoint

Achieving target improvement in lifestyle risk factors at 12 weeks and at 1 year. Target improvement will be considered if the patient achieves any one or more of the following:

1. Smoking cessation
2. BMI 20–25 (kg/m^2). BMI is calculated by dividing body weight in kilogrammes by the square of height in meters
3. HbA1c less than 7%
4. Total cholesterol less than 5.0 mmol/L

Patients will be reassessed for the primary endpoint at 1 year. If they fail the criteria by which they were deemed at 12 weeks to have achieved the primary endpoint, they will then be considered to not have achieved the 1-year primary endpoint.

We will report the primary endpoint for both time points.

#### Secondary endpoints

Secondary endpoints of PAD outcomes are based on the Society for Vascular Surgery (SVS) reporting standards [17]:

1. Amputation-free survival; defined as time spent free from any major above ankle amputation [17]
2. Hemodynamic improvement: defined as an increase in the ABI by at least 0.10 [17]
3. Clinical improvement: which is defined as an upward shift by at least one Rutherford category. However, PAD patients suffering from actual tissue loss (Rutherford category 5 [19]) should move up two Rutherford categories to be considered improved [19].
4. Re-intervention or stenosis rate; any re-intervention or stenosis among patients who already underwent vascular surgery [17]
5. Freedom from major adverse cardiovascular events (MACE). MACE is defined as any major cardiovascular event such as myocardial infarction, cerebrovascular accident or death [17].
6. Major adverse limb events (MALE). MALE is defined as any major amputation or revascularization procedure [17].
7. Revascularisation-free survival; defined as time free from any revascularisation procedure regardless of if it were an endovascular intervention or an open surgery [17].
8. Change from baseline of the following risk factors:
  a- BMI (measured in kg/m^2^)
  b- HbA1c (measured as a percentage)
  c- Total cholesterol (measured in mmol/L)
9. Health-related quality of life [18]; assessed using the Dartmouth Cooperative Information Project (COOP) charts at enrolment and after 1 year. The COOP charts measure six core aspects of functional status: physical fitness, feelings, daily activities, social activities, change in health, pain, and overall health. The instrument consists of six charts, referring to the abovementioned aspects of functioning. Each chart consists of a simple title, a question referring to the status of the patient and an ordinal 5-point response scale illustrated with a simple drawing. Each item is rated on this 5-point ordinal scale ranging from 1 (no limitation at all) to 5 (severely limited); for “change in health” score 1 means “much better” and score 5 “much worse”. The designers do not advocate summing the responses to gain a single index figure of health status

#### Safety endpoints

1. Incidence and severity of adverse events
2. Incidence of side effects due to medication commenced during the trial, for the modulation of any PAD risk factor. This includes any drug interaction of these medications with any previously assumed medication the patient was using regularly prior to commencing the trial.

### Sample size calculation

For sample size calculation, the EUROACTION study [30] was used to estimate the coefficient of variation for sample proportions.

Data from the EUROACTION [30] study suggest that 12-week intervention response rates for the primary endpoint of 54.8% (intervention programme) and 35.6% (usual care). Eighty percent statistical power and an alpha level of 5% were chosen. A two-sample comparison of proportions sample size calculation was implemented.

With these parameters, the G*Power [31] software yields a trial with a maximum sample size of 208 patients completing the intervention (104 per intervention group).

### Statistical analysis

All data will be analysed according to the intention to treat principle. The primary outcome, the achievement of treatment goals for PAD risk factors between both groups at 12 weeks and 12 months will be compared using chi-square or Fisher’s exact where appropriate. Secondary outcomes including time to event will be assessed using Kaplan-Meier survival curves and log rank test. An exact 95% confidence interval will be applied for the difference between intervention groups in terms of PAD risk factor reduction.

## Discussion

PAD is a very common disease that affects the quality of life of a large segment of the global population [1]. While PAD has been associated with increased risk of cardiovascular events, yet most recommendations for risk factor modulation in this population has been based mainly on studies on patients with cardiovascular disease [14–16, 32].

These recommendations for lifestyle modification techniques in PAD patients have included smoking cessation based on level 1B evidence and healthy diet and physical activity based on level 1C evidence [12]. However, none of these recommendations was based on randomised clinical trials. In fact, the recommendation for smoking cessation in PAD patients was based on observational studies that noted that smokers had a seven-fold increased risk of developing PAD [33, 34] and a two-fold higher risk of amputation [35]. There are no randomised controlled trials that have quantified the direct effect of smoking cessation, directly targeting this specific PAD population.

It is worth noting that most PAD patients have multifactorial risk factors. This adds to the limitations of any study designed to target risk factor modulation. Studies designed with a single risk factor treatment approach have the potential to fail, due to the confounding effects of other risk factors. Alternatively, in a study like our proposed trial, where a full multi-risk factor improvement approach is adopted, it is difficult to assess, which particular intervention yielded the best outcome.

In a multifactorial risk factor modification programme, some of the risk factors, like blood pressure, blood tests, anthropometrics and exercise testing assessments, are easily assessed with objective measurement tools. However, certain aspects can only be assessed using subjective methods like the HADS [26], Dartmouth Quality of Life score [18], Fagerstrom Test for Nicotine Dependence [27] and Mediterranean Diet Questionnaire [29]. This is a potential limitation of this study, due to the inherent bias of patients responding to such subjective questionnaires.

It has been shown that patients with PAD are routinely undertreated for most risk factors [4], which may contribute to high rates of morbidity and mortality. However, in the absence of level 1 evidence to support its implementation in this particular cohort of patients, it could be difficult to convince health authorities of the benefits of spending on such programmes. Cost of providing a multifactorial risk factor modification programme to all PAD patients could be prohibitive. However, if proven to be effective and included in the guidelines of management of PAD patients, this added cost should be weighed against the potential benefits and long-term savings of reducing cardiovascular events. We aim with this study to provide clear evidence for the effectiveness of a lifestyle and risk factor modification intervention programme in achieving treatment goals for PAD risk factors, through a high-quality, well-powered clinical trial.

Any important protocol modifications will be communicated first to the REC. Following ethical approval, an amended patient information leaflet will be circulated to trial participants. The trial registries and journal will be notified of the amended protocol.

### Trial registration

This trial was registered (11/07/2017) on the European Clinical Trials Database (EudraCT number 2017-002964-41) and ClinicalTrials.gov (NCT03935776) which was registered on 02 May 2019. Additional file 1; Section 1, shows Trial Registration Data Set.

### Trial status

The study is ongoing at the time of submitting this manuscript (November 2019). This trial was using protocol version 2.0 (14 March 2018) at the time of this submission. Recruitment started in the University College Hospital, Galway, Ireland on 1 June 2018 and is expected to be completed on 1 June 2021. The trial management committee manages and disseminates the protocol amendments.

## Supplementary Information

**Additional file 1:.** Section 1. Trial Registration Data Set. Section 2. Consent Form for the RCT. Section 3. Consent Form Checklist for the RCT.

## Data Availability

The final datasets underlying publications, resulting from this trial, will be shared as an anonymous copy upon reasonable and approved request. A request may be made through email to the Principal Investigator and can only be made upon meeting the terms and conditions for the ethics approval of this trial.

## Abbreviations

ABI: Ankle-brachial index
AE: Adverse event
BMI: Body mass index
BRPM: Blinded report planning meeting
CRF: Case report form
CTA: Clinical Trial Authorisation
FAS: Full Analysis (data) Set
HADS: Hospital Anxiety and Depression Scale
HbA1c: Glycosylated haemoglobin
HDL: High-density lipoprotein
ICH: International Conference on Harmonisation
LDL: Low-density lipoprotein
MACE: Major adverse cardiovascular event
MALE: Major adverse limb event
MDT: Multidisciplinary team
NUI: National University of Ireland, Galway
PAD: Peripheral arterial disease
PP: Per protocol
SAE: Serious adverse event
SAP: Statistical analysis plan
SVS: Society for Vascular Surgery
UCHG: University College Hospital, Galway, Ireland
WIfI: Wound ischaemia and foot infection classification
