# Supplementary material for: The effect of lifestyle and risk factor modification on occlusive peripheral arterial disease outcomes: standard healthcare vs structured programme—for a randomised controlled trial protocol
Source: Trials. 2021 Feb 13;22:138. doi: 10.1186/s13063-021-05087-x (PMC7881542; doi:10.1186/s13063-021-05087-x)

**Section 1.** Trial Registration Data Set.

| **Data Category** | **Information** |
| --- | --- |
| Primary registry and trial identifying number | ClinicalTrials.gov (NCT03935776) |
| Date of registration in primary registry | 02 May 2019 |
| Secondary identifying numbers | WVI-PAD |
| Source(s) of monetary or material support | Galway University Hospital |
| Primary sponsor | Ministry of Higher Education and Scientific Research, Libyan Embassy |
| Secondary sponsor(s) | NA |
| Contact for public queries | ME, marahelfghi@gmail.com |
| Contact for scientific queries | ME, marahelfghi@gmail.com |
| Public title | Risk Factor Modification Programme for Peripheral Arterial Disease Patients |
| Scientific title | The Effect of Lifestyle and Risk Factor Modification on Occlusive Peripheral Arterial Disease Outcomes: Standard Healthcare vs Structured Programme: for a Randomised Controlled Trial Protocol |
| Countries of recruitment | Republic of Ireland |
| Health condition(s) or problem(s) studied | Occlusive Peripheral Arterial Disease |
| Intervention(s) | -Risk Factor Modification Structured Programme  - Standard healthcare |
| Key inclusion and exclusion criteria | Inclusion criteria: Ages eligible for study: ≥18years; symptomatic PAD and at least one PAD risk factors.  Exclusion criteria: Asymptomatic PAD; involvement in another clinical trial in the previous six months; legal incapacity; inadequate English language; significant cognitive impairment or mental illness; refusal to participate in a certain part of the intervention; immobility and contraindication to anticoagulation and antiplatelet medications or any of the risk factors treatment |
| Study type | Treatment |
| Date of first enrolment | 1 June 2018 |
| Target sample size | 208 patients |
| Recruitment status | Recruiting |
| Primary outcome(s) | Achieving one or more target Improvement in lifestyle risk factors. Which includes: Smoking cessation; BMI 20-25 (kg/m^2); HbA1c less than 7% and total Cholesterol less than 5.0 mmol/L |
| Key secondary outcomes | PAD outcomes are based on the Society for Vascular Surgery (SVS) reporting standards^20^: These include: Amputation free survival; any intervention, above-ankle amputation, or stenosis; re-intervention or above knee amputation; freedom from MACE and MALE; revascularisation-free survival; health related quality of life. |

**Section 2**. Consent Form for the RCT.


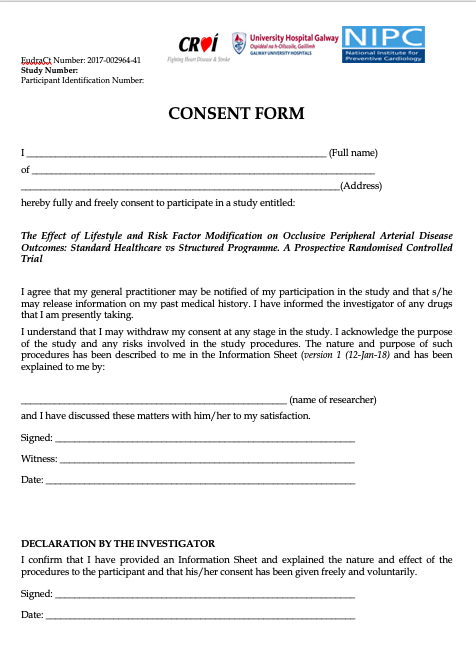


**Section 3.** Consent Form Checklist for the RCT


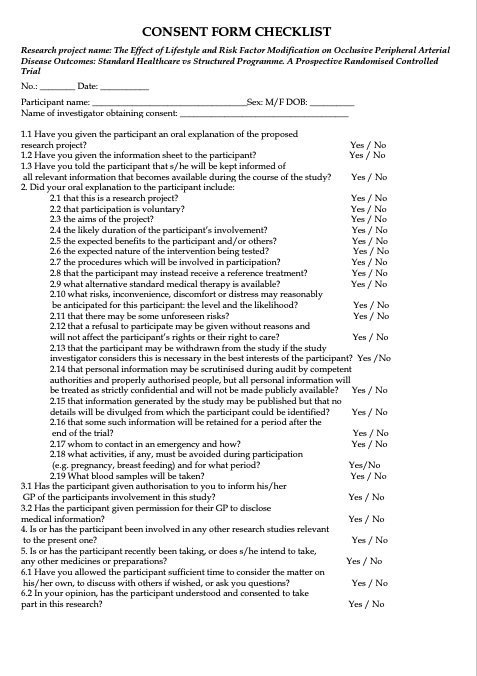

Supplement: Supplementary file 1 — Additional file 1:. Section 1. Trial Registration Data Set. Section 2. Consent Form for the RCT. Section 3. Consent Form Checklist for the RCT. [file 13063_2021_5087_MOESM1_ESM.docx]
